# Ethanolic Extract of *Sophora moorcroftiana* Seeds Attenuates LPS-Induced Inflammation and Oxidative Stress in RAW 264.7 Macrophages via Modulation of the p62/Keap1/Nrf2 Signaling Pathway

**DOI:** 10.3390/antiox15080974

**Published:** 2026-08-05

**Authors:** Nianshou Zhao, Hongya Li, Peng Ji, Yanming Wei, Yongli Hua, Yanan Guo, Fanlin Wu

**Affiliations:** 1 College of Veterinary Medicine, Gansu Agricultural University, Lanzhou 730070, China; 2 Institute of Animal Science, Ningxia Academy of Agriculture and Forestry, Yinchuan 750002, China; 3 Lanzhou Institute of Husbandry and Pharmaceutical Science, Chinese Academy of Agricultural Science, Lanzhou 730050, China

**Keywords:** *Sophora moorcroftiana* seeds, anti-inflammatory activity, antioxidant activity, reactive oxygen species, p62/Keap1/Nrf2 signaling pathway

## Abstract

Despite the traditional application of *Sophora moorcroftiana* seeds (SMS) in Tibetan medicine for heat-clearing, damp-drying, anti-inflammatory and detoxifying properties, the anti-inflammatory mechanisms of SMS remain insufficiently understood and warrant systematic investigation. This study evaluated the protective effects of an ethanolic SMS extract against lipopolysaccharide (LPS)-induced oxidative stress and inflammation in RAW 264.7 macrophages, with emphasis on the p62/Keap1/Nrf2 signaling pathway. In vitro, the extract exhibited significant DPPH, ABTS and ·OH radical scavenging activities, as well as ferric-reducing antioxidant power, in a clear concentration dependent manner. An inflammatory model was established by stimulating RAW 264.7 cells with LPS, followed by treatment with graded concentrations of the ethanolic SMS extract. The extract significantly reduced LPS-induced nitric oxide production and decreased the secretion of TNF-α, IL-6 and IL-1β, while suppressing iNOS and COX-2 expression at both mRNA and protein levels. In parallel, the extract alleviated oxidative stress, as evidenced by reduced intracellular reactive oxygen species (ROS) and MDA levels, increased antioxidant defenses including SOD, GSH and CAT, and decreased LDH release. At the protein-expression level, SMS treatment was accompanied by differential changes in p62, Keap1, total Nrf2 and HO-1 expression, suggesting that its effects may involve regulatory processes associated with cellular stress and antioxidant defense. Collectively, the ethanolic extract of SMS attenuated LPS-induced inflammatory and oxidative stress responses in RAW 264.7 macrophages. These effects may be associated with the suppression of inflammatory mediator production, reduction in the cellular oxidative stress burden and modulation of proteins involved in cellular stress responses and antioxidant defense.

## 1. Introduction

Inflammation and oxidative stress are tightly interconnected. Inflammatory mediators promote oxidative stress and oxidative stress in turn amplifies inflammation through feed-forward signaling mechanisms [1]. As a core innate immune response to tissue injury, infection, or external stimuli, inflammation is essential for host protection and tissue repair [2]. While a controlled inflammatory response helps eliminate harmful triggers, persistent or dysregulated activation can escalate into a cytokine storm and contribute to chronic inflammatory diseases, such as rheumatoid arthritis, multiple sclerosis and type 1 diabetes [3,4]. When inflammation persists or endogenous antioxidant defenses decline, reactive oxygen species (ROS) accumulate abnormally and impair cellular function. By oxidatively modifying macromolecules including carbohydrates, proteins, lipids and DNA, ROS reinforce a self-perpetuating cycle of inflammation and oxidative damage, thereby aggravating disease progression [5,6].

Macrophages are key immune effector cells and occupy a central role in inflammatory regulatory networks [7]. Upon stimulation, activated macrophages produce inflammatory mediators, including cyclooxygenase 2 (COX-2) and inducible nitric oxide synthase (iNOS) and secrete pro-inflammatory cytokines, such as tumor necrosis factor alpha (TNF-α), interleukin 1 beta (IL-1β) and interleukin 6 (IL-6) [8]. Notably, iNOS catalyzes the generation of high levels of nitric oxide (NO) in specific tissue microenvironments, and excessive NO can contribute to oxidative stress-mediated injury. Activated inflammatory cells are also a major source of ROS. When ROS production exceeds the capacity of endogenous antioxidant defenses, oxidative imbalance disrupts the structure and function of cellular macromolecules and accelerates disease progression [9]. In addition, ROS can directly activate inflammatory signaling pathways, promoting the cascade release of pro-inflammatory mediators and further amplifying the inflammatory response [10].

The Keap1/Nrf2 signaling pathway is a crucial antioxidant pathway. By maintaining equilibrium between the oxidative and antioxidant systems, this pathway helps cells defend against the harmful effects of oxidative stress [11]. Nrf2 is a core transcription factor that regulates cellular defense mechanisms in response to environmental stress [12]. Under steady-state conditions, Nrf2 is maintained at low levels in the cytoplasm through its interaction with Keap1 [13]. During oxidative stress, the Nrf2/Keap1 interaction is dissociated in a dose-dependent manner. Free and newly synthesized Nrf2 translocates to the nucleus, binds to Maf heterodimers, and activates the antioxidant response element [14]. Initiating downstream gene transcription to mediate antioxidant, detoxification, anti-inflammatory, autophagy and proteasome-related cytoprotective effects [15]. Additionally, Nrf2 activity is regulated by autophagy flux and the selective autophagy receptor p62. Under conditions of autophagy impairment or oxidative stress, p62 interacts with Keap1 and promotes its selective autophagic degradation, thereby releasing Nrf2 from inhibition and enhancing the expression of Nrf2 dependent antioxidant defense genes [16]. Therefore, suppressing inflammation and oxidative stress may be an effective strategy for alleviating acute immune responses.

*Sophora moorcroftiana* (SM), is a perennial dwarf shrub belonging to the Fabaceae family. Primarily distributed in the Yarlung Tsangpo Valley, it serves as a key community-forming shrub species in the arid and semi-arid riverine zones of this area. Its seeds (*Sophora moorcroftiana seeds, SMS*), as one of Tibet’s traditional medicinal herbs, are commonly used in folk medicine for conditions such as jaundice-related hepatitis and damp-heat jaundice due to its properties of clearing heat, drying dampness, detoxifying and reducing swelling. It is also rich in various chemical constituents including alkaloids, flavonoids, esters and steroids [17]. Alkaloids are often considered the primary active constituents, associated with anti-inflammatory, antioxidant, antitumor and immunomodulatory effects [18,19]. Our research group has previously established HPLC-based qualitative and quantitative methods for the analysis of bioactive constituents in the ethanolic extract of SMS, identifying oxymatrine as the primary constituent [20]. Oxymatrine demonstrates significant therapeutic efficacy in combating oxidative stress, reducing inflammation, preventing atherosclerosis, restoring vascular function and inhibiting cardiac remodeling and failure [21]. Although the pharmacological activity of SMS has garnered some attention, the mechanisms underlying its key targets and signaling pathways for anti-inflammatory effects remain to be further elucidated.

In the present study, RAW 264.7 macrophages were used as an in vitro model to systematically evaluate the anti-inflammatory activity of the ethanolic extract of SMS, with particular emphasis on its regulation of the p62/Keap1/Nrf2 signaling axis and the underlying molecular mechanisms. This work is expected to provide molecular evidence supporting the traditional use of SMS, offering a theoretical basis and experimental rationale for developing bioactive constituents from Tibetan medicine for use in treating inflammation-related diseases.

## 2. Materials and Methods

### 2.1. Reagents and Material

RAW 264.7 macrophages were purchased from Wuhan Pricella Biotechnology Co., Ltd. (Wuhan, China) (Database Name: Cellosaurus; Accession Numbers: CVCL_0493). Fetal bovine serum (FBS) (Cat. No. DCF-201-0500) was purchased from Dcell Biologics (Shanghai, China); DMEM/F-12 (Cat. No. G4612-500 mL), penicillin-streptomycin solution (Cat. No. BL505A) and cell counting kit-8 (CCK-8) (Cat. No. BS350C) were purchased from Biosharp (Beijing, China). A nitric oxide assay kit (Cat. No. S0033M) was purchased from Beyotime Biotechnology (Shanghai, China). Lipopolysaccharide (LPS) (L8080) was purchased from Solarbio (Beijing, China). 2,2-diphenyl-1-picrylhydrazyl (DPPH) (CAS: 1898-66-4), 2,4,6-tripyridyl-s-triazine (TPTZ) (CAS: 3682-35-7) and 2,2-azino-bis-(3-ethyl-benzthia-6-sulfonic acid) (ABTS) (CAS: 30931-67-0) were purchased from Shanghai Macklin Biotech Co., Ltd. (Shanghai, China). Mouse IL-6 (Cat. No. RXW203049M), mouse IL-1β (RX203063M), mouse TNF-α (Cat. No. RXW202412M) and mouse IL-10 (Cat. No. RX203075M) kits were obtained from Quanzhou Ruixin Biotechnology Co., Ltd. (Quanzhou, China). SOD (A001-3-2), GSH (Cat. No. A006-2-1), MDA (Cat. No. A003-1-2) and LDH (Cat. No. A003-1-2) assay kits were purchased from Nanjing Jiancheng Bioengineering Institute (Nanjing, China). TransZol Up (Cat. No. ET111-01-V2), PerfectStart Visual Green qPCR SuperMix (Cat. No. CM0139) and TransScript^®^ All-in-One First-Strand cDNA Synthesis Supermix for qPCR (Cat. No. AG11728) were purchased from TransGen Biotech (Beijing, China). Recombinant rabbit monoclonal antibodies against COX-2 (Cat. No. ET1610-23), iNOS (Cat. No. HA722031), p62 and HRP-conjugated goat anti-mouse IgG polyclonal antibody (Cat. No. HA1006) were purchased from Hangzhou HuaAn Biotechnology Co., Ltd. (Hangzhou, China). Recombinant rabbit polyclonal antibody against Keap1 (Cat. No. 10503-2-AP) and mouse monoclonal antibody against HO-1 (Cat. No. 66743-1-Ig) were obtained from Proteintech Group, Inc. (Wuhan, China). Recombinant rabbit polyclonal antibody targeting GAPDH (Cat. No. bs-10900R) and HRP-conjugated goat anti-rabbit IgG H&L (Cat. No. bs-0295G-HRP) were supplied by Beijing Biosynthesis Biotechnology Co., Ltd. (Beijing, China). Mouse monoclonal antibody against Nrf2 (clone A-10, Cat. No. sc-365949) was purchased from Santa Cruz Biotechnology, Inc. (Shanghai, China) and recombinant mouse monoclonal antibody against β-actin (Cat. No. GB12001-100) was acquired from Wuhan Servicebio Technology Co., Ltd. (Wuhan, China). Reactive oxygen species (ROS) assay kit (Cat. No. S0033S) was purchased from Beyotime Biotechnology (Shanghai, China). All other chemical reagents and solvents were of analytical grade.

### 2.2. Preparation of the Ethanolic Extract of SMS

SMS were collected in Shigatse, Tibet, China, and authenticated by the Department of Traditional Chinese Veterinary Medicine, College of Veterinary Medicine, Gansu Agricultural University. After the removal of impurities, the seeds were crushed and passed through a 40-mesh sieve. The ethanolic extract prepared according to a protocol previously optimized in our laboratory [20]. Briefly, the powdered seeds were extracted with 75% ethanol at a solid-to-liquid ratio of 1:30 g/mL. The suspension was subjected to ultrasound-assisted extraction at 55 °C for 50 min with a tan output power of 150 W.

### 2.3. Effects of the Ethanolic Extract of SMS on RAW 264.7 Cell Viability

RAW 264.7 macrophages were maintained in DMEM/F-12 supplemented with 10% FBS and 1% penicillin-streptomycin at 37 °C in a humidified incubator with 5% CO_2_. Cells in the logarithmic growth phase were seeded into 96-well plates at a density of 1 × 10^4^ cells per well and allowed to adhere for 24 h. The culture medium was then replaced with a fresh medium containing the ethanolic extract of SMS at final concentrations of 0, 50, 100, 200, 400, 800, 1000, 2000 and 3000 µg/mL. After incubation for 6, 12 and 24 h, the medium was removed and basal medium containing 10% CCK-8 reagent was added to each well. The plates were incubated for a further 1.5 h, and absorbance was measured at 450 nm using a microplate reader to evaluate cell viability.

### 2.4. Effect of Varying LPS Concentrations on NO Levels in RAW 264.7 Macrophages

RAW 264.7 macrophages in the logarithmic growth phase were seeded into 24-well plates at a density of 1 × 10^5^ cells per well and incubated for 24 h at 37 °C in a humidified incubator with 5% CO_2_. The culture medium was then replaced with fresh medium containing LPS at final concentrations of 0.25, 0.5, 1.0 and 2.0 µg/mL. After 6, 12 and 24 h of stimulation, the culture supernatants were collected into centrifuge tubes and centrifuged at 4 °C for 5 min. The NO levels in the clarified supernatants were then determined using a nitric oxide assay kitaccording to the manufacturer’s instructions.

### 2.5. Effects of the Ethanolic Extract of SMS on NO Production and Cell Morphology in RAW 264.7 Macrophages

RAW 264.7 macrophages in the logarithmic growth phase were seeded into 24-well plates at a density of 2 × 10^5^ cells per well and incubated for 24 h at 37 °C in a humidified incubator with 5% CO_2_. The culture medium was then replaced. Cells in the control and LPS groups received fresh basal medium, whereas cells in the SMS groups were pretreated with a medium containing the ethanolic extract of SMS at final concentrations of 50, 200 and 800 µg/mL. After 6 h of pretreatment, all groups except the control were stimulated with LPS at a final concentration of 0.25 µg/mL and incubated for a further 24 h. Cell morphology was subsequently observed under an inverted microscope. The culture supernatants were then collected and centrifuged at 4 °C for 5 min, and NO production was determined using a nitric oxide assay kit according to the manufacturer’s instructions.

### 2.6. Scavenging Activity of the Ethanolic Extract of SMS on DPPH, ABTS, ·OH Radicals and Ferric-Reducing Antioxidant Power (FRAP)

#### 2.6.1. DPPH Radical Scavenging Activity Assay

The DPPH radical scavenging activity of the ethanolic extract of SMS was evaluated using a previously described method with slight modifications [22]. Initially, a wide concentration range was set for preliminary screening. Based on the results of the preliminary experiment, the concentration range of 2.4–10,000 μg/mL was determined for the formal test. Serial dilutions of the extract within the above concentration range were prepared using 60% ethanol. Aliquots of 100 μL of each dilution were mixed with 100 μL of DPPH solution (0.2 mM in anhydrous ethanol) in 96-well plates. The mixtures were gently shaken and incubated at room temperature (25 ± 2 °C) in the dark for 30 min. The absorbance of the sample (Abs. sample) was measured at 517 nm using a microplate reader. The absorbance of the control (Abs. control), in which anhydrous ethanol was used instead of DPPH solution, and the absorbance of the blank (Abs. blank), in which 60% ethanol was used instead of the test sample, were also recorded. Ascorbic acid (VC) was used as a positive control, and all measurements were performed in triplicate.

DPPH scavenging activity (%) = [1 − ((Abs. sample − Abs. control)/Abs. blank)] × 100

#### 2.6.2. ABTS Radical Scavenging Activity Assay

The ABTS radical scavenging activity of the ethanolic extract of SMS was evaluated using a previously described method with slight modifications [22]. Initially, a wide concentration range was set for preliminary screening. Based on the results of the preliminary experiment, the concentration range of 19.5–8000 μg/mL was determined for the formal test. Serial dilutions of the extract within the above concentration range were prepared using 60% ethanol. The ABTS stock solution was prepared by mixing 7 mM ABTS with 2.45 mM K_2_S_2_O_8_ at a 1:1 (*v*/*v*) ratio and allowing the mixture to stand in the dark for 16 h at room temperature. Before use, the stock solution was diluted with 60% ethanol to obtain an absorbance of 0.70 ± 0.05 at 734 nm. Aliquots of 20 μL of each sample dilution were added to 180 μL of the ABTS working solution in 96-well plates and vortexed thoroughly. The reaction mixtures were incubated at room temperature (25 ± 2 °C) in the dark for 10 min, and the absorbance of the samples (Abs. sample) was measured at 734 nm. The absorbance of the control (Abs. control), in which deionized water was used instead of the ABTS working solution, and the absorbance of the blank (Abs. blank), in which 60% ethanol was used instead of the test sample, were also recorded. VC was used as a positive control, and all measurements were performed in triplicate.

ABTS scavenging activity (%) = [1 − ((Abs. sample − Abs. control)/Abs. blank)] × 100

#### 2.6.3. ·OH Radical Scavenging Activity Assay

The ·OH radical scavenging activity of the ethanolic extract of SMS was determined using a previously described method with minor modifications [23]. Initially, a wide concentration range was set for preliminary screening. Based on the results of the preliminary experiment, the concentration range of 78.1–5000 μg/mL was determined for the formal test. Serial dilutions of the extract within the above concentration range were prepared using 60% ethanol. Aliquots of 150 μL of each sample solution were mixed with 25 μL of 1.5 mM FeSO_4_, 20 μL of 20 mM salicylic acid and 20 μL of 6 mM H_2_O_2_. The mixtures were gently shaken and incubated at 37 °C in the dark for 1 h. The absorbance of the samples (Abs. sample) was then measured at 520 nm. The absorbance of the control (Abs. control), in which deionized water was used instead of H_2_O_2_, and the absorbance of the blank (Abs. blank), in which 60% ethanol was used instead of the test sample, were also recorded. VC was used as a positive control, and all measurements were performed in triplicate.

·OH scavenging activity (%) = [1 − ((Abs. sample − Abs. control)/Abs. blank)] × 100

#### 2.6.4. FRAP Assay

FRAP of SMS ethanolic extract was measured using the previously reported spectrophotometric method with slight modifications [24]. Initially, a wide concentration range was set for preliminary screening. Based on the results of the preliminary experiment, the concentration range of 19.531–2500 μg/mL was determined for the formal test. Serial dilutions of the extract within the above concentration range were prepared using 60% ethanol. Aliquots of 50 μL of each sample solution were mixed with 150 μL of freshly prepared FRAP reagent, consisting of 300 mM acetate buffer at pH 3.6, 10 mM TPTZ in 40 mM HCl and 20 mM FeCl_3_·6H_2_O solution in a 10:1:1 (*v*/*v*/*v*) ratio. The mixtures were incubated at room temperature (25 ± 2 °C) for 8 min, and the absorbance of the samples (Abs. sample) was measured at 593 nm. The blank (Abs. blank) was prepared by replacing the sample solution with deionized water. VC was used as a positive control, and all measurements were performed in triplicate. A standard curve was generated using FeSO_4_ solutions prepared from a 2 mmol/L stock solution by serial dilution and measured under the same conditions.

FRAP (μmol/L) = (Abs. sample − Abs. blank)/Slope of FeSO_4_ standard curve

### 2.7. Effect of the Ethanolic Extract of SMS on Inflammatory Cytokines and Oxidative Stress Markers in LPS-Induced RAW 264.7 Macrophages

RAW 264.7 macrophages were treated as described in Section 2.5, and culture supernatants from each group were collected. The concentrations of the inflammatory cytokines TNF-α, IL-1β, IL-6 and IL-10, as well as the levels of the oxidative stress markers GSH and MDA and the activities of LDH and SOD, were quantified using commercial Elisa and assay kits in accordance with the manufacturers’ instructions.

### 2.8. Effect of the Ethanolic Extract of SMS on LPS-Induced mRNA Expression of Inflammatory Cytokines and Oxidative Stress Markers in RAW 264.7 Macrophages

RAW 264.7 macrophages in the logarithmic growth phase were seeded into 12-well plates at a density of 5 × 10^5^ cells per well and incubated for 24 h at 37 °C in a humidified incubator with 5% CO_2_. The culture medium was replaced with fresh basal medium for the control and LPS groups, while cells in the SMS groups were pretreated with a medium containing the ethanolic extract of SMS at concentrations of 50, 200 and 800 µg/mL for 6 h. After pretreatment, all groups except the control were stimulated with 0.25 µg/mL LPS and incubated for an additional 24 h. Subsequently, the culture medium was aspirated, and the cells were washed twice with PBS. Total RNA was extracted using a Trizol reagent kit, and complementary DNA (cDNA) was synthesized via reverse transcription using the Evo M-MLV reverse transcription kit. Real-time quantitative polymerase chain reaction (RT-qPCR) was performed using the synthesized cDNA as the template. The amplification protocol was as follows: initial denaturation at 95 °C for 30 s, followed by 45 cycles of denaturation at 95 °C for 5 s, and combined annealing/extension at 60 °C for 30 s. The relative mRNA expression levels were quantified using the 2^–ΔΔCt^ method. The primer sequences used are listed in Table 1.

### 2.9. Effect of the Ethanolic Extract of SMS on ROS Levels in LPS-Induced RAW 264.7 Macrophages Using DCFH‑DA Fluorescent Probe

RAW 264.7 macrophages in the logarithmic growth phase were seeded into 12-well plates at a density of 2 × 10^5^ cells per well and incubated for 24 h at 37 °C in a humidified incubator with 5% CO_2_. The control and LPS groups were replenished with fresh basal medium, while the control and treatment groups were pretreated with fresh medium containing ethanolic extract of SMS for 6 h. After pretreatment, all groups except the control and ethanolic extract of SMS control groups were stimulated with 0.25 µg/mL LPS and incubated for an additional 24 h. The culture medium was then aspirated, and the cells were washed once with basal medium. DCFH-DA (1:1000), a fluorescent probe, was added to the cells and incubated in the dark for 30 min. Following incubation, the probe-containing medium was removed, and the cells were washed three times with basal medium. The fluorescence intensity of each group was recorded and captured using an inverted fluorescence microscope.

### 2.10. Effect of the Ethanolic Extract of SMS on the p62/Keap1/Nrf2 Signaling Pathway in LPS-Induced RAW 264.7 Macrophages

Following the grouping described in Section 2.8, the culture supernatant was removed and the cells were washed twice with PBS 250 μL of RIPA lysis buffer was added to each well, and the cells were lysed on ice for 20 min. The lysates were centrifuged at 14,000 *g* for 5 min at 4 °C, and the supernatants were collected. Protein samples were mixed with 4× loading buffer and denatured by boiling at 100 °C for 10 min, then separated via sodium dodecyl sulfate-polyacrylamide gel electrophoresis (SDS-PAGE). Following electrophoresis, the intact gel was assembled together with a 0.45 μm polyvinylidene fluoride (PVDF) membrane and subjected to simultaneous wet transfer within the same transfer tank to ensure uniform transfer conditions and parameters across all samples. Post-transfer, PVDF membranes were blocked with 5% skim milk at room temperature for 2 h before incubation with diluted primary antibodies. Membranes were then incubated overnight at 4 °C on a shaking incubator using the primary antibodies listed below: COX-2 (1:1000), iNOS (1:1000), p62 (1:1000), Nrf2 (1:2000), Keap1 (1:2000), HO-1 (1:3000), GAPDH (1:3000) and β-actin (1:3000). After washing, the membrane was incubated with horseradish peroxidase-conjugated rabbit secondary antibody (1:10,000) and mouse secondary antibody (1:8000) for 1 h at room temperature. Finally, the protein bands were visualized using an enhanced chemiluminescence substrate and detected with a gel imaging system (Amersham ImageQuant™ 800 Western blot Imaging System, Cytiva, Marlborough, MA, USA). Band grayscale values were analyzed using ImageJ 1.8.0 software.

### 2.11. Statistical Analysis

All data were analyzed using IBM SPSS Statistics version 26. The results are expressed as the mean ± standard deviation (SD). Statistical significance was determined by one-way ANOVA followed by Tukey’s honestly significant difference test. Bars with different letters indicate significant differences (*p* < 0.05).

## 3. Results and Discussion

### 3.1. Qualitative and Quantitative Analysis of Active Components in the Ethanolic Extract of SMS

**Figure 1:**
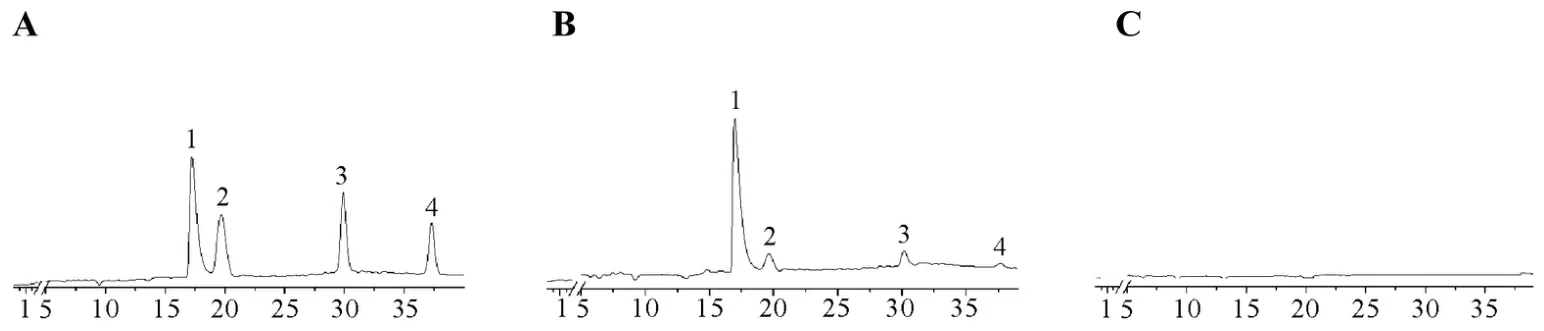
HPLC chromatograms used for the qualitative identification and quantitative determination of major bioactive constituents in the ethanolic extract of SMS. (**A**) Mixed standard; (**B**) the ethanolic extract of SMS sample; (**C**) negative control; 1 oxymatrine; 2 oxysophocarpine; 3 matrine; 4 sophocarpine.

Our research group has previously performed HPLC-based qualitative identification and quantitative determination of the major bioactive constituents in the ethanolic extract of SMS (Figure 1). The results showed that the extract mainly contained oxymatrine, oxysophocarpine, matrine and sophocarpine. Among these, oxymatrine was the most abundant and was identified as the predominant active component in the extract [20]. Previous studies have confirmed that matrine, oxymatrine and sophocarpine all exhibit significant antioxidant and anti-inflammatory activities. Matrine can enhance the activities of GSH-PX and CAT in H9C2 cardiomyocytes injured by hyperglycemia or H_2_O_2_, reduce the release of inflammatory factors such as IL-1β, IL-6 and TNF-α by inhibiting the NF-κB pathway, and alleviate myocardial oxidative stress and inflammatory responses. Oxymatrine downregulates the expression of autophagy-related proteins LC3II/LC3I, Beclin-1 and Atg5, decreases the levels of TNF-α, IL-6 and IL-8, and maintains the homeostatic balance of autophagy in cardiomyocytes [21]. Sophocarpine significantly ameliorates myocardial oxidative stress and apoptosis, reduces total ROS and MDA content, increases SOD and GSH levels, inhibits pro-apoptotic proteins including Bax, cleaved caspase-3 and cytochrome c via activating the Nrf2/HO-1 pathway, and upregulates Bcl-2 expression, thereby mitigating myocardial injury [25]. The ethanolic extract of SMS contains active components such as matrine, oxymatrine and sophocarpine, among which oxymatrine is the most abundant, suggesting that it is highly likely to be the key material basis for the anti-inflammatory and antioxidant effects of SMS.

### 3.2. Effect of the Ethanolic Extract of SMS on the Viability of RAW 264.7 Macrophages

**Figure 2:**
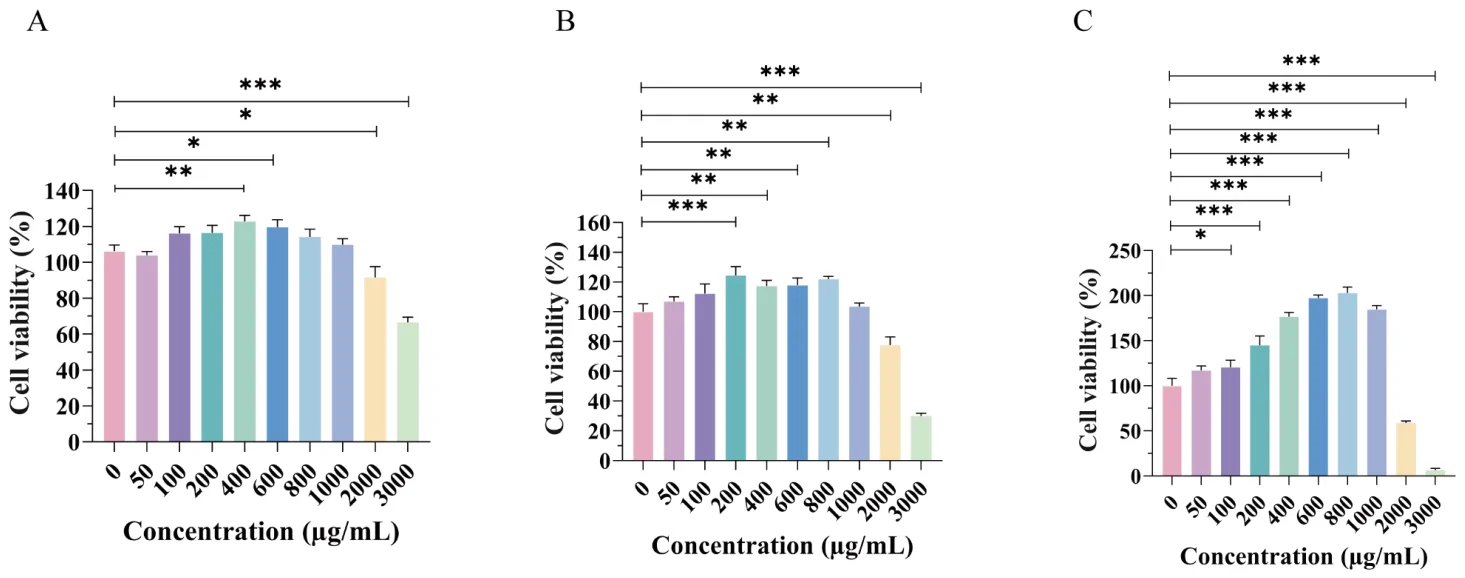
Effect of the ethanolic extract of SMS on RAW 264.7 cell viability (*n* = 6). (**A**) Cell viability after 6 h treatment; (**B**) cell viability after 12 h treatment; (**C**) cell viability after 24 h treatment. All data are expressed as mean ± SD. * *p* < 0.05, ** *p* < 0.01 and *** *p* < 0.001 indicate significant differences compared with the group without the ethanolic extract of SMS.

The ethanolic extract of SMS exhibited dose and time dependent effects on the viability of RAW 264.7 macrophages (Figure 2). After a 6 h treatment with concentration ranging from 50 to 800 µg/mL, cell viability increased gradually with rising extract concentrations. When the treatment duration was extended to 12 and 24 h, cell viability was significantly higher compared to the 6 h time point. Notably, after 24 h of treatment at 800 µg/mL, cell viability was 2.02 times that of the control group. These results indicated that the ethanolic extract of SMS exhibited no obvious cytotoxicity toward RAW 264.7 macrophages at concentrations ranging from 50 to 800 µg/mL, with favorable biological safety. Moreover, it significantly enhanced cell viability, which helped maintain normal cellular physiological functions and laid a foundation for the subsequent regulation of inflammatory responses. Considering both safety and effective working concentrations comprehensively, three concentrations of 50, 200 and 800 µg/mL were selected for subsequent experiments in this study.

### 3.3. Anti-Inflammatory Effects of the Ethanolic Extract of SMS in LPS-Induced RAW 264.7 Macrophages

#### 3.3.1. Effect of the Ethanolic Extract of SMS on NO Production in the Supernatant of RAW 264.7 Macrophages

**Figure 3:**
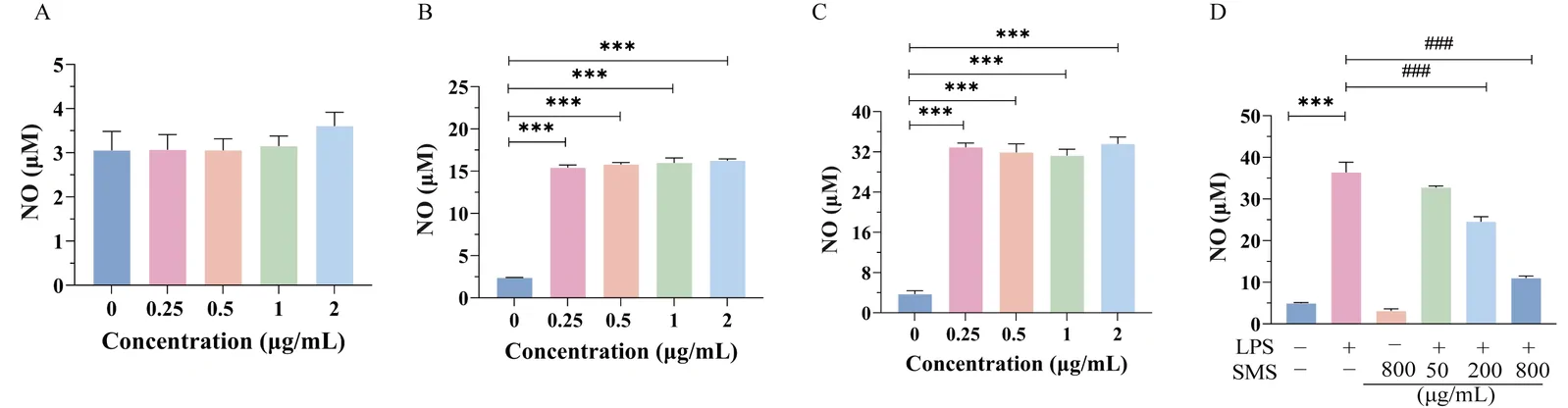
Effect of the ethanolic extract of SMS on LPS-induced NO release in RAW 264.7 macrophages (*n* = 4). (**A**) NO levels in cells treated with different concentrations of LPS for 6 h; (**B**) NO levels in cells treated with different concentrations of LPS for 12 h; (**C**) NO levels in cells treated with different concentrations of LPS for 24 h; (**D**) effect of different concentrations of the ethanolic extract of SMS on NO levels induced by LPS after 24 h. −, without corresponding intervention/stimulation; +, with corresponding intervention/stimulation. All data are expressed as mean ± SD. *** *p* < 0.001 indicates significant differences compared with the LPS-untreated control group; ^###^ *p* < 0.001 indicates significant differences compared with the LPS-induced group.

NO is an important inflammatory mediator produced by activated macrophages. In activated macrophages, sustained and excessive NO production is closely associated with increased iNOS expression and may contribute to the maintenance and amplification of inflammatory responses, thereby aggravating oxidative damage [25,26]. In the present study, NO production in RAW 264.7 macrophages was significantly increased after 12 and 24 h of LPS stimulation (Figure 3B,C). However, no significant difference in NO levels was observed between the 0.25 and 2 μg/mL LPS groups, indicating that 0.25 μg/mL LPS was sufficient to induce a marked increase in NO production and that further increasing the LPS concentration did not enhance this response. Therefore, considering both the inflammatory response and the LPS concentration, stimulation with 0.25 μg/mL LPS for 24 h was selected to establish the inflammatory cell model for subsequent experiments. Following pretreatment with 50, 200 and 800 μg/mL SMS ethanolic extract for 6 h, LPS-induced NO production was reduced to varying degrees and generally decreased with increasing SMS concentration, with the greatest reduction observed at 800 μg/mL. In contrast, treatment with the SMS ethanolic extract alone did not increase NO levels, indicating that SMS did not directly stimulate NO production in RAW 264.7 macrophages within the tested concentration range (Figure 3D). Previous studies have similarly shown that natural alkaloids, including matrine and sophocarpine can suppress LPS-induced NO production in RAW 264.7 macrophages [27], which is consistent with the present findings. Given that excessive NO production is an important component of the LPS-induced macrophage inflammatory response, the inhibitory effect of SMS on NO production may contribute to the attenuation of macrophage inflammatory activation.

#### 3.3.2. Effect of the Ethanolic Extract of SMS on the Morphology of RAW 264.7 Macrophages

**Figure 4:**
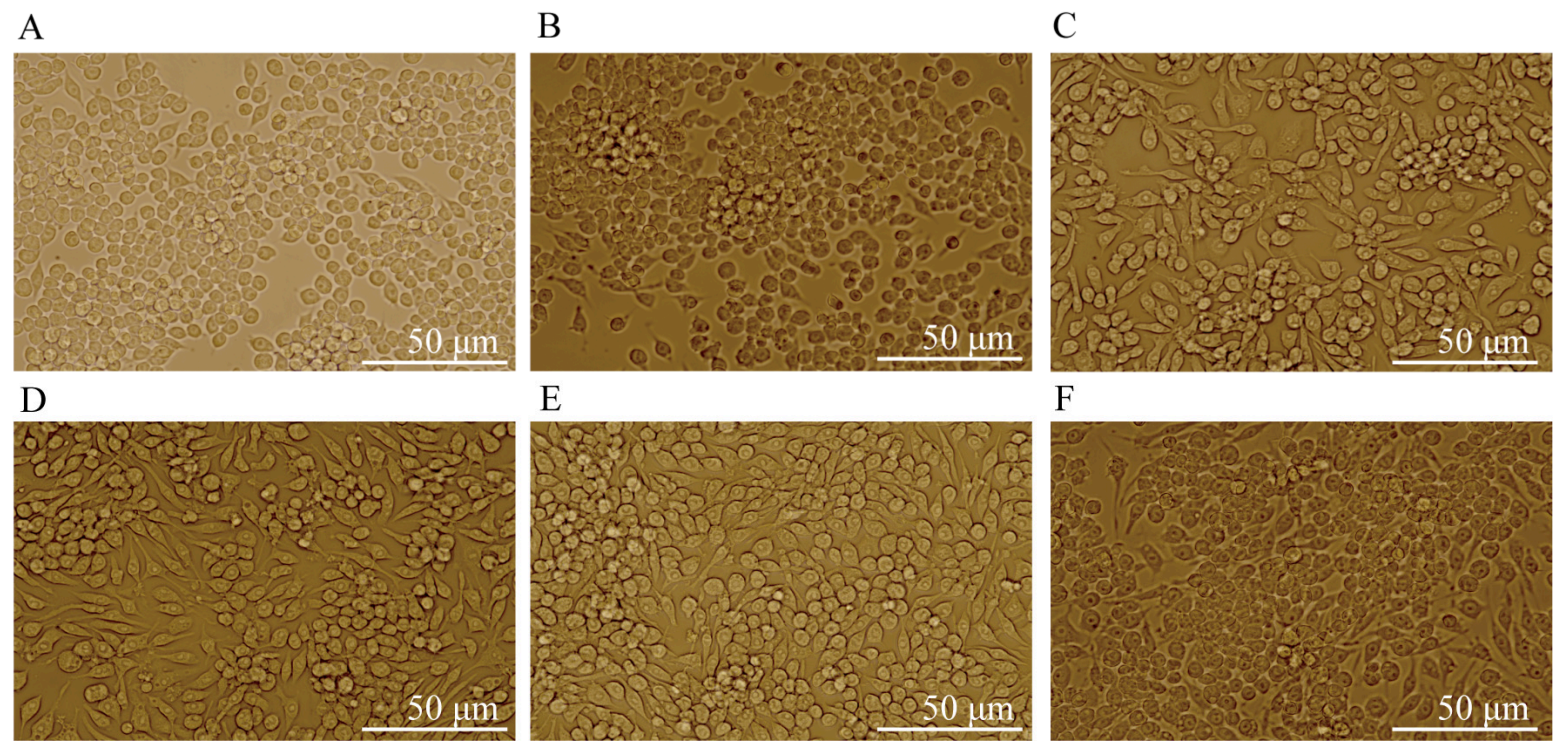
Morphological effects of the ethanolic extract of SMS on LPS-induced RAW 264.7 macrophages. (**A**) Control group; (**B**) 800 μg/mL ethanolic extract of SMS group; (**C**) LPS-induced group; (**D**) LPS + 50 μg/mL ethanolic extract of SMS group; (**E**) LPS + 200 μg/mL ethanolic extract of SMS group; (**F**) LPS + 800 μg/mL ethanolic extract of SMS group.

LPS stimulation can induce marked morphological remodeling in macrophages, characterized primarily by cell spreading, elongation of the cell body, and the formation of pseudopodia and other cellular protrusions. These changes generally reflect macrophage activation in response to inflammatory stimulation [28,29]. In the present study, untreated RAW 264.7 macrophages and cells treated with the SMS ethanolic extract alone were predominantly round. Following LPS stimulation, the cells became markedly elongated or spindle-shaped and developed prominent pseudopodia. Pretreatment with 50, 200 and 800 μg/mL SMS ethanolic extract for 6 h attenuated these LPS-induced morphological changes to varying degrees after 24 h of stimulation, with the morphology of cells in the 800 μg/mL group most closely resembling that of the control group (Figure 4). Previous studies have likewise shown that LPS stimulation induces a transition of RAW 264.7 macrophages from a round morphology to irregular or elongated shapes, accompanied by increased cell spreading and pseudopodia formation. Intervention with anti-inflammatory agents partially attenuated these morphological alterations and was accompanied by reductions in NO production or iNOS expression [30,31]. The effects of SMS pretreatment on cellular morphology and NO production observed in the present study were broadly consistent with these findings, suggesting that SMS can attenuate LPS-induced inflammatory activation in RAW 264.7 macrophages.

#### 3.3.3. Effect of the Ethanolic Extract of SMS on the Levels of TNF-α, IL-1β, IL-6 and IL-10 in LPS-Induced RAW 264.7 Macrophages

**Figure 5:**
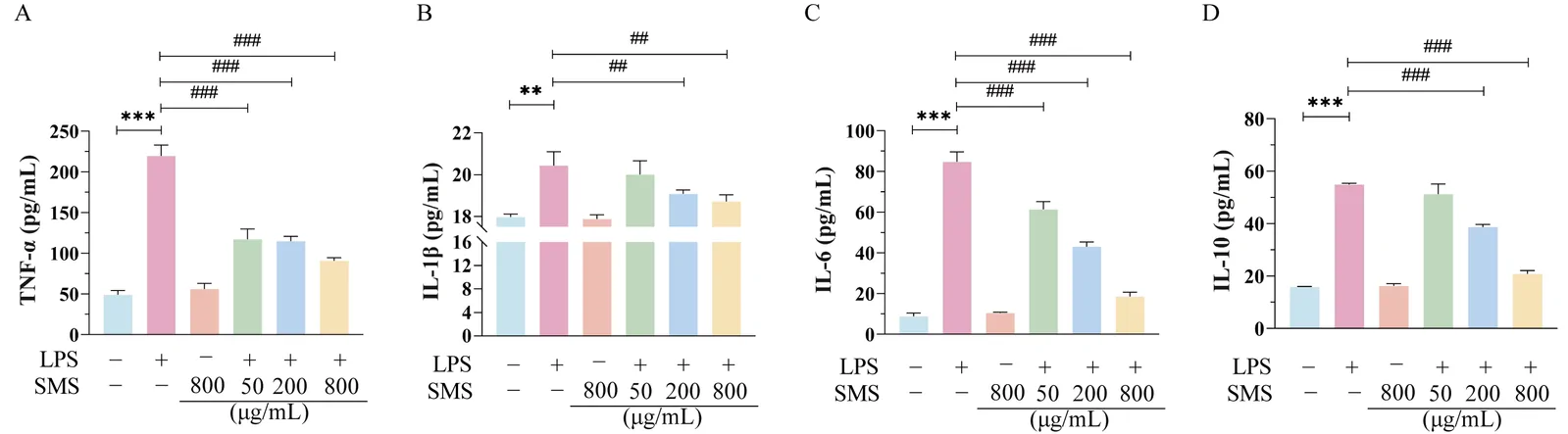
Effect of the ethanolic extract of SMS on the expression of inflammatory cytokines in LPS-induced RAW 264.7 macrophages (*n* = 4). (**A**) TNF-α; (**B**) IL-1β; (**C**) IL-6; (**D**) IL-10. −, without corresponding intervention/stimulation; +, with corresponding intervention/stimulation. All data are expressed as mean ± SD. ** *p* < 0.01 and *** *p* < 0.001 indicate significant differences compared with the LPS-untreated control group; ^##^ *p* < 0.01 and ^###^ *p* < 0.001 indicate significant differences compared with the LPS-induced group.

Cytokines are important signaling molecules involved in immune regulation and inflammatory responses. Following inflammatory stimulation, macrophages release pro-inflammatory cytokines such as TNF-α, IL-1β and IL-6, which contribute to the initiation and maintenance of inflammation. However, excessive or sustained production of these cytokines may disrupt inflammatory homeostasis and aggravate tissue injury [32,33]. Therefore, limiting the excessive release of pro-inflammatory cytokines is an important aspect of controlling macrophage-mediated inflammatory responses [34]. In the present study, LPS stimulation for 24 h significantly increased the concentrations of TNF-α, IL-1β and IL-6 in the culture supernatant of RAW 264.7 macrophages, indicating a marked pro-inflammatory cytokine response. Pretreatment with 50, 200 and 800 μg/mL SMS ethanolic extract for 6 h resulted in an overall concentration-related reduction in these cytokines, with the greatest decreases observed in the 800 μg/mL group (Figure 5). These findings indicate that SMS attenuated LPS-induced pro-inflammatory cytokine production in RAW 264.7 macrophages. Notably, LPS stimulation also significantly increased IL-10 levels in the culture supernatant, whereas SMS pretreatment reduced LPS-induced IL-10 secretion. IL-10 is an important immunoregulatory cytokine produced by macrophages following LPS stimulation and can limit the sustained amplification of TLR4-associated pro-inflammatory signaling through negative-feedback regulation [35,36]. Therefore, the increase in IL-10 following LPS stimulation may represent a compensatory response to the enhanced pro-inflammatory state. Previous studies have similarly shown that inhibition of LPS-induced inflammatory signaling may reduce IL-1β and IL-6 production while also being accompanied by a decrease in IL-10 levels [37]. In the present study, the reductions in TNF-α, IL-1β and IL-6 following SMS pretreatment were accompanied by a decrease in IL-10, which was broadly consistent with these observations. This response pattern suggests that SMS may attenuate the overall LPS-induced cytokine response, thereby reducing both pro-inflammatory mediator production and the compensatory IL-10 secretion elicited by the inflammatory response.

#### 3.3.4. Effect of the Ethanolic Extract of SMS on the Expression of Inflammation-Related Genes in LPS-Induced RAW 264.7 Macrophages

**Figure 6:**
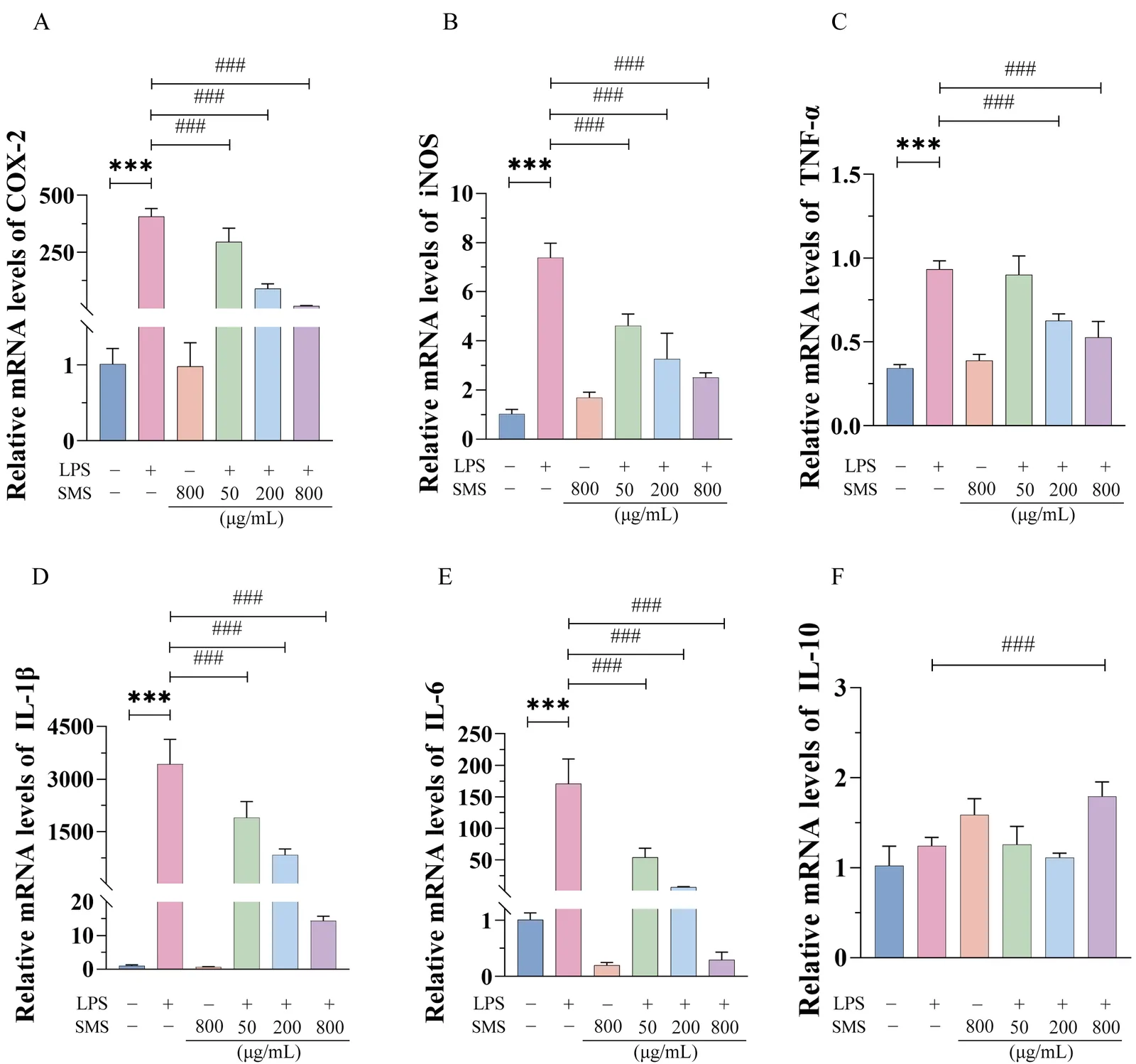
Effect of the ethanolic extract of SMS on the mRNA expression of inflammatory cytokines in LPS-induced RAW 264.7 macrophages (*n* = 4). (**A**) COX-2 mRNA; (**B**) iNOS mRNA; (**C**) TNF-α mRNA; (**D**) IL-1β mRNA; (**E**) IL-6 mRNA; (**F**) IL-10 mRNA. −, without corresponding intervention/stimulation; +, with corresponding intervention/stimulation. All data are expressed as mean ± SD. *** *p* < 0.001 indicates significant differences compared with the LPS-untreated control group; ^###^ *p* < 0.001 indicates significant differences compared with the LPS-induced group.

LPS stimulation of RAW 264.7 macrophages induces the expression of inflammation-related enzymes, including COX-2 and iNOS, as well as pro-inflammatory cytokines such as TNF-α, IL-1β and IL-6 [32,33,38]. These molecules contribute to the initiation and maintenance of inflammatory responses, whereas their sustained or excessive expression may disrupt inflammatory regulation and further amplify inflammation [39]. In the present study, LPS stimulation significantly upregulated the mRNA expression of COX-2, iNOS, TNF-α, IL-1β and IL-6 in RAW 264.7 macrophages. Pretreatment with 50, 200 and 800 μg/mL SMS ethanolic extract suppressed the expression of these genes to varying degrees, with an overall concentration-related downward trend and the greatest reductions observed in the 800 μg/mL group (Figure 6). Notably, IL-10 exhibited a distinct response pattern. LPS stimulation did not significantly alter IL-10 mRNA expression but significantly increased IL-10 protein levels in the culture supernatant. Pretreatment with 800 μg/mL SMS further increased IL-10 mRNA expression while reducing its extracellular protein level, indicating that IL-10 transcript abundance and secreted protein production did not change synchronously. Time-course studies in RAW 264.7 macrophages have shown that LPS-induced IL-10 transcription and protein secretion follow different temporal patterns. Consequently, measurements of mRNA and extracellular protein at a single time point may reflect different phases of the cellular response [40]. In addition, IL-10 production is regulated at multiple post-transcriptional and secretory levels, including mRNA stability, translational efficiency, intracellular trafficking and secretion [41,42,43]. Previous studies have also demonstrated that IL-10 protein production in LPS-stimulated macrophages can be regulated without corresponding changes in promoter activity or mRNA abundance, further supporting a degree of dissociation between IL-10 transcription and protein production [44]. Therefore, the increase in IL-10 mRNA accompanied by a decrease in extracellular IL-10 protein observed in the present study may be related to temporal differences between transcription and secretion, as well as regulation at post-transcriptional or secretory stages. Because time-course analyses, intracellular IL-10 protein measurements, mRNA stability assays and secretory trafficking assessments were not performed, the precise mechanisms underlying this discrepancy remain to be clarified. With the exception of IL-10, the effects of SMS on inflammation-related gene expression were generally consistent with the corresponding inflammatory measurements. SMS pretreatment downregulated LPS-induced COX-2, iNOS, TNF-α, IL-1β and IL-6 mRNA expression and reduced the levels of NO, TNF-α, IL-1β and IL-6 in the culture supernatant. The broadly concordant changes at the transcriptional and extracellular levels suggest that the SMS ethanolic extract suppresses LPS-induced inflammatory gene expression and inflammatory mediator production, thereby attenuating inflammatory activation in RAW 264.7 macrophages.

#### 3.3.5. Effect of the Ethanolic Extract of SMS on the COX-2 and iNOS Protein Expression in LPS-Induced RAW 264.7 Macrophages

**Figure 7:**
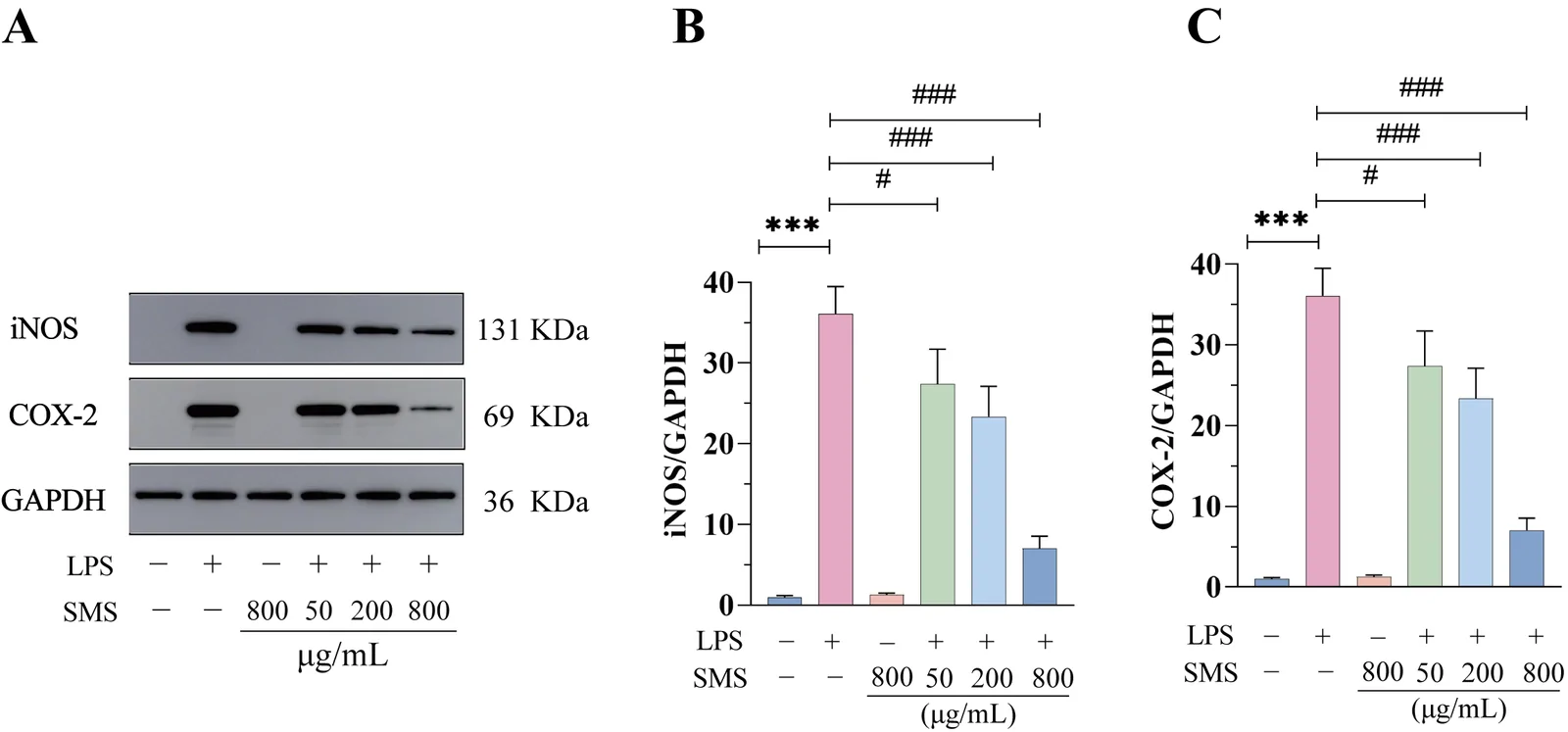
Effect of the ethanolic extract of SMS on the expression of iNOS and COX-2 proteins in LPS-induced RAW 264.7 macrophages (*n* = 3). (**A**) COX-2 and iNOS protein expression; (**B**) quantitative analysis of iNOS protein expression; (**C**) quantitative analysis of COX-2 protein expression. −, without corresponding intervention/stimulation; +, with corresponding intervention/stimulation. All data are expressed as mean ± SD. *** *p* < 0.001 indicates significant differences compared with the LPS-untreated control group; ^#^ *p* < 0.05 and ^###^ *p* < 0.001 indicate significant differences compared with the LPS-induced group.

In resting macrophages, iNOS and COX-2 are generally expressed at low levels. Following LPS stimulation, both enzymes can be rapidly induced and contribute to the production of inflammatory mediators. iNOS primarily catalyzes NO synthesis, whereas COX-2 participates in the biosynthesis of prostanoid mediators under inflammatory conditions. Therefore, both are commonly used as indicators of macrophage inflammatory activation [45]. In the present study, LPS stimulation significantly increased iNOS and COX-2 protein expression in RAW 264.7 macrophages, whereas pretreatment with the SMS ethanolic extract attenuated these changes to varying degrees, with the greatest reductions observed in the 800 μg/mL group (Figure 7). Previous studies have shown that sophocarpine and other natural bioactive compounds can downregulate iNOS and COX-2 expression in LPS-stimulated RAW 264.7 macrophages, accompanied by reductions in NO and other inflammatory mediators [46,47]. The present findings were broadly consistent with these reports. When considered together with the preceding mRNA results, the effects of SMS on iNOS and COX-2 were directionally consistent at both the transcriptional and protein levels. This indicates that the regulation of these inflammation-related effector enzymes by SMS was not confined to a single level of expression but involved in the concordant suppression of both mRNA and protein expression. The reduction in iNOS protein expression corresponded with the decrease in NO levels in the culture supernatant, suggesting that the inhibitory effect of SMS on NO production may be associated with iNOS down regulation. Meanwhile, both COX-2 mRNA and protein expression were markedly reduced. Together with the concurrent decreases in NO, TNF-α, IL-1β and IL-6, these findings provide convergent evidence at the levels of gene transcription, protein expression and inflammatory mediator production that SMS attenuates LPS-induced inflammatory activation in RAW 264.7 macrophages.

### 3.4. Effect of the Ethanolic Extract of SMS on the Antioxidant Activity in LPS-Induced RAW 264.7 Macrophages

#### 3.4.1. In Vitro Antioxidant Activity of the Ethanolic Extract of SMS

**Figure 8:**
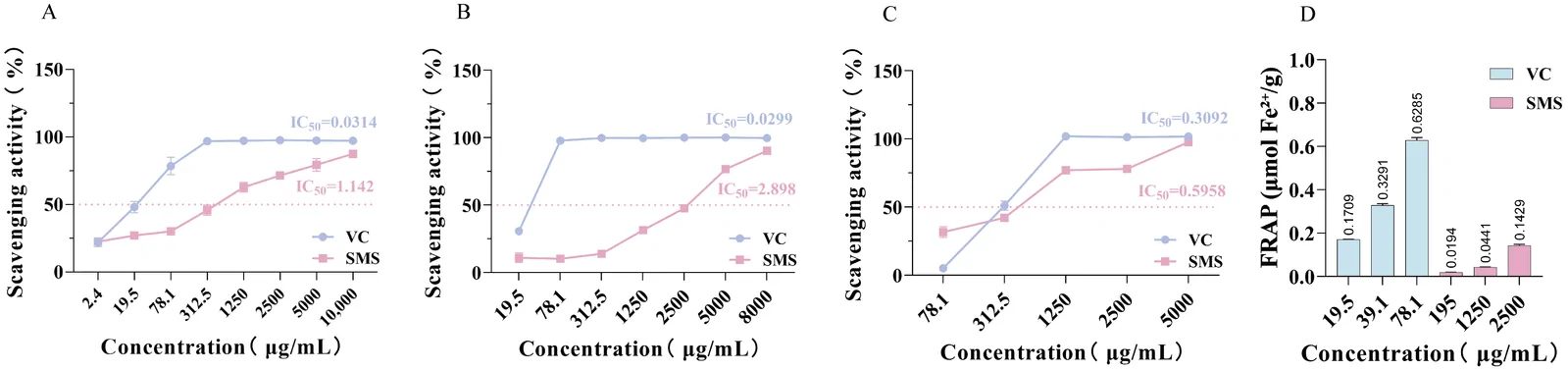
In vitro free radical scavenging activity of the ethanolic extract of SMS (n = 3). (**A**) DPPH free radical scavenging activity; (**B**) ABTS free radical scavenging activity; (**C**) OH free radical scavenging activity; (**D**) ferric-reducing antioxidant power. The dotted horizontal line in the figure indicates the 50% scavenging‑activity threshold for IC_50_ determination.

Excessive free radicals can induce oxidative stress, interfere with normal metabolic functions, and contribute to various diseases and tissue damage [48]. Among the diverse methods employed to assess antioxidant activity, scavenging assays for DPPH, ABTS and ·OH, along with the FRAP assay, are the most commonly employed techniques [49]. In this study, the ethanolic extract of SMS exhibited varying degrees of scavenging activity against DPPH, ABTS and ·OH radicals, and demonstrated significant reducing power in the FRAP assay, with a clear concentration-effect relationship between its scavenging activity and extract concentration (Figure 8). The half-maximal inhibitory concentrations (IC_50_) for DPPH, ABTS and ·OH were 1142, 2898 and 595.8 µg/mL, respectively. At a concentration of 10,000 µg/mL, the DPPH scavenging activity of the ethanolic extract of SMS approached that of VC (Figure 8A). At 8000 µg/mL, the ABTS scavenging activity approached that of VC (Figure 8B). At 5000 µg/mL, the ·OH scavenging activity approached that of VC (Figure 8C). Furthermore, in the FRAP assay, at 2500 µg/mL, the FRAP value of the ethanolic extract of SMS was comparable to that of VC, with a reducing power of 0.1429 μmol Fe^2+^/g (Figure 8D). These results further confirmed in vitro that the ethanolic extract of SMS possesses significant antioxidant activity in vitro. It exerts antioxidant effects by directly scavenging multiple free radicals including DPPH, ABTS and ·OH, and enhancing reducing power, and this activity shows an obvious concentration-dependent manner.

#### 3.4.2. Effect of the Ethanolic Extract of SMS on Oxidative Stress Markers in LPS-Induced RAW 264.7 Macrophages

**Figure 9:**
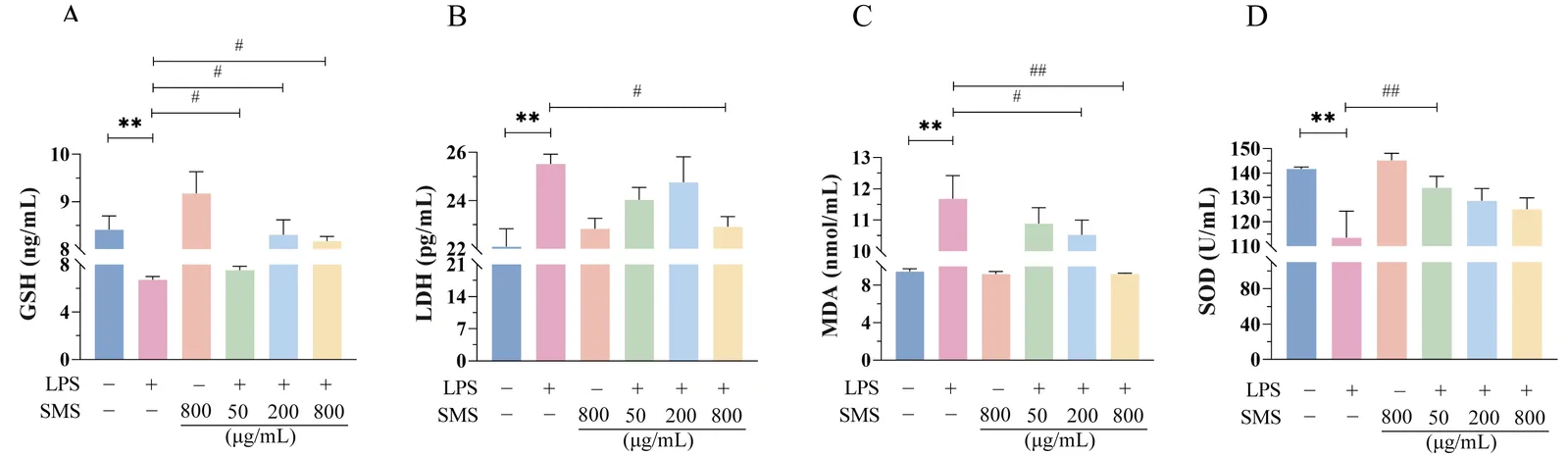
Effect of the ethanolic extract of SMS on oxidative stress markers in LPS-induced RAW 264.7 macrophages (*n* = 4). (**A**) GSH; (**B**) LDH; (**C**) MDA; (**D**) SOD. −, without corresponding intervention/stimulation; +, with corresponding intervention/stimulation. All data are expressed as mean ± SD. ** *p* < 0.01 indicates significant differences compared with the LPS-untreated control group; ^#^ *p* < 0.05 and ^##^ *p* < 0.01 indicate significant differences compared with the LPS-induced group.

When the body is stimulated to produce ROS, cell membrane phospholipids generate large amounts of malondialdehyde (MDA), which inhibits the activity of superoxide dismutase (SOD) and glutathione (GSH) [50]. The excessive accumulation of MDA further disrupts mitochondrial function and exacerbates cell membrane damage [51]. Meanwhile, inhibition of SOD and GSH activity leads to the excessive accumulation of superoxide anions, intensifying oxidative stress and triggering inflammatory damage [52]. Lactate dehydrogenase (LDH), a soluble cytoplasmic enzyme, is considered an important marker of cell injury when released into the extracellular space. In this study, LPS stimulation significantly inhibited the activity of GSH and SOD in RAW 264.7 macrophages and promoted the release of LDH and MDA. However, following pretreatment with the SMS ethanolic extract for 6 h and subsequent LPS stimulation, the activity of GSH and SOD was significantly enhanced, and the levels of MDA and LDH were significantly reduced. Within the concentration range of 50–800 µg/mL, the ethanolic extract of SMS showed a concentration-dependent effect on enhancing the activity of GSH and SOD, as well as inhibiting LDH and MDA levels, with the 800 µg/mL group showing the most significant effect (Figure 9). These results further verified the antioxidant activity of the ethanolic extract of SMS from the perspective of cellular oxidative damage repair. It not only exerts in vitro antioxidant effects by directly scavenging free radicals, but also activates the endogenous antioxidant system, enhances the activities of key antioxidant enzymes such as SOD and GSH, reduces the accumulation of lipid peroxidation product MDA, alleviates cell membrane damage and LDH release, and prevents the aggravation of oxidative stress.

#### 3.4.3. Effect of the Ethanolic Extract of SMS on the mRNA Expression of Oxidative Stress Markers in LPS-Induced RAW 264.7 Macrophages

**Figure 10:**
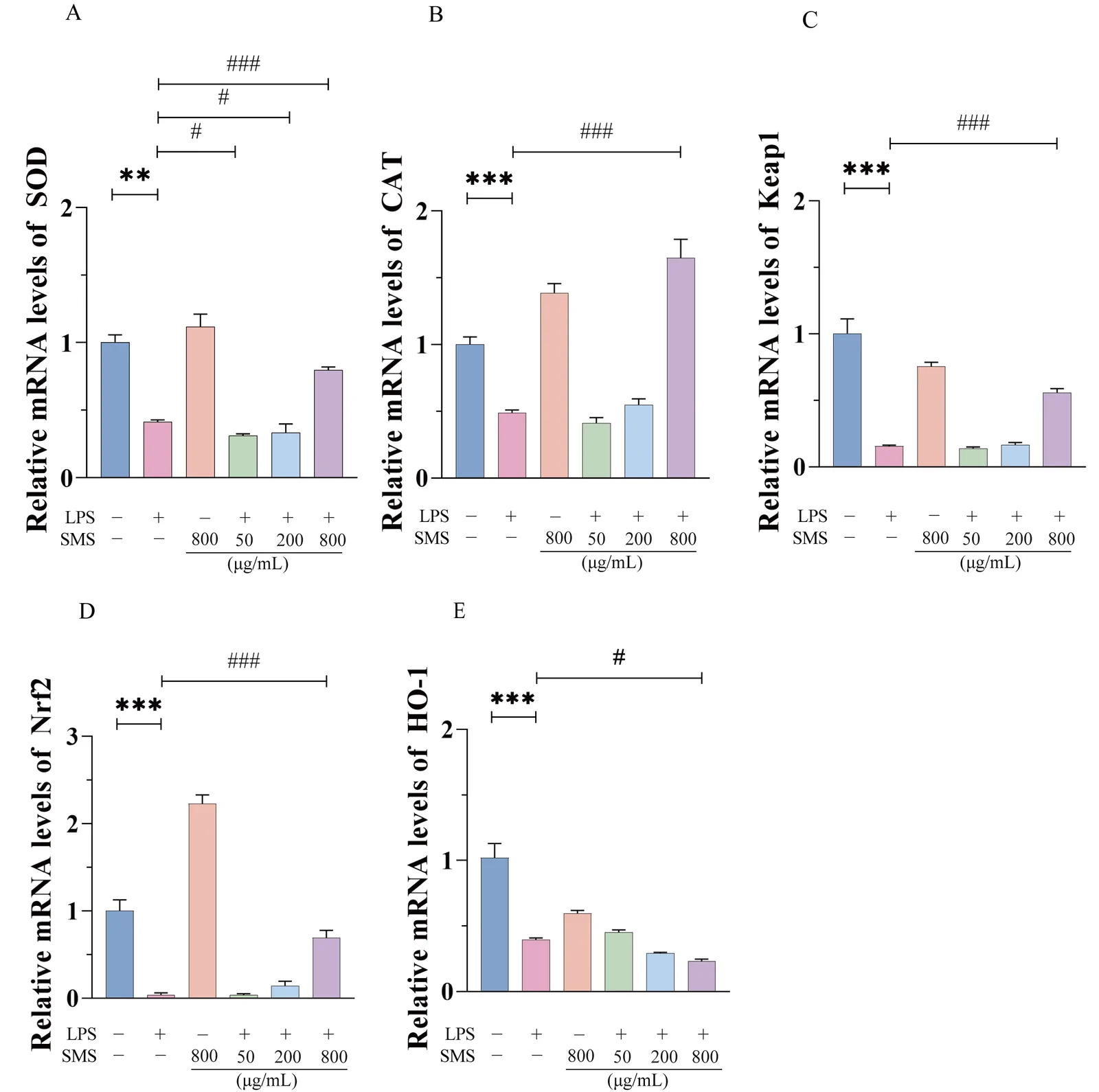
Effect of the ethanolic extract of SMS on the mRNA expression of oxidative stress markers in LPS-induced RAW 264.7 macrophages (*n* = 4). (**A**) SOD mRNA; (**B**) CAT mRNA; (**C**) Keap1 mRNA; (**D**) Nrf2 mRNA; (**E**) HO-1 mRNA. −, without corresponding intervention/stimulation; +, with corresponding intervention/stimulation. All data are expressed as mean ± SD. ** *p* < 0.01 and *** *p* < 0.001 indicate significant differences compared with the LPS-untreated control group; ^#^ *p* < 0.05 and ^###^ *p* < 0.001 indicate significant differences compared with the LPS-induced group.

Catalase (CAT) and SOD are important components of the cellular antioxidant defense system [53]. SOD catalyzes the conversion of superoxide anions into hydrogen peroxide, which is subsequently eliminated by CAT and other antioxidant enzymes, thereby limiting ROS accumulation and the resulting cellular damage [54,55]. In the present study, LPS stimulation significantly reduced SOD and CAT mRNA expression in RAW 264.7 macrophages, whereas pretreatment with the SMS ethanolic extract for 6 h increased the expression of both genes to varying degrees. These findings indicate that SMS partially alleviated the LPS-induced suppression of antioxidant-related gene transcription. SMS treatment also partially restored the LPS-suppressed Nrf2 mRNA level. However, HO-1 mRNA did not exhibit a similar response and instead decreased further with increasing SMS concentrations (Figure 10), indicating that SMS exerted nonuniform effects on different antioxidant-related genes. Nrf2 activity is regulated primarily through Keap1-mediated protein degradation, protein stability and nuclear translocation, whereas p62 can influence Nrf2 stability and accumulation through its interaction with Keap1 [56,57]. Therefore, partial restoration of Nrf2 mRNA expression does not necessarily indicate enhanced Nrf2 transcriptional activity, because the expression of its downstream genes is also influenced by Nrf2 protein stability, nuclear translocation and other transcriptional regulators. Although HO-1 is an important antioxidant-related gene regulated by Nrf2, its transcription is also controlled by repressors such as BACH1. Effective induction of HO-1 depends not only on Nrf2 but also on the dissociation of BACH1 from the corresponding regulatory regions [58]. Thus, the partial recovery of Nrf2 mRNA accompanied by a continued decrease in HO-1 mRNA may reflect differences in their transcriptional regulation and expression kinetics. In addition, the effects of Nrf2 on macrophage inflammatory responses are not entirely dependent on HO-1. Previous studies have shown that Nrf2 can also restrict the transcription of pro-inflammatory genes such as IL-6 and IL-1β. Together with the increased SOD and CAT mRNA expression, partial restoration of Nrf2 mRNA, and reductions in NO, TNF-α, IL-1β and IL-6 following SMS treatment, these findings suggest that the SMS ethanolic extract may modulate LPS-induced oxidative stress-related transcriptional responses and inflammation. The divergent changes in Nrf2 and HO-1 mRNA further indicate that the effects of SMS on antioxidant-related genes were not fully coordinated and may involve gene-specific transcriptional regulation and distinct expression kinetics.

#### 3.4.4. Effect of the Ethanolic Extract of SMS on ROS Levels in LPS-Induced RAW 264.7 Macrophages

**Figure 11:**
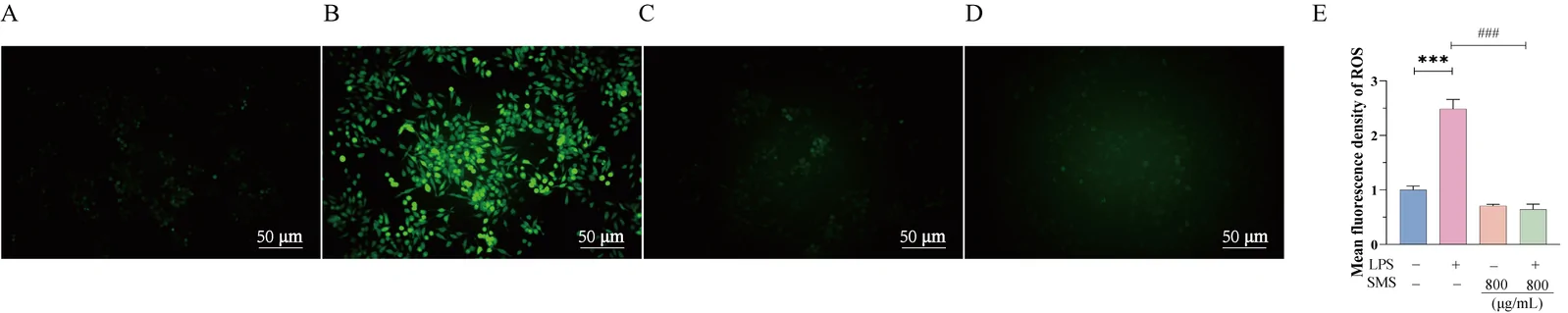
Effect of the ethanolic extract of SMS on ROS generation in LPS-induced RAW 264.7 macrophages (*n* = 4). (**A**) Control group; (**B**) LPS group; (**C**) 800 μg/mL ethanolic extract of SMS group; (**D**) LPS + 800 μg/mL ethanolic extract of SMS group; (**E**) quantitative analysis of ROS. −, without corresponding intervention/stimulation; +, with corresponding intervention/stimulation. All data are expressed as mean ± SD. *** *p* < 0.001 indicates significant differences compared with the LPS-untreated control group; ^###^ *p* < 0.001 indicates significant differences compared with the LPS-induced group.

ROS are important reactive molecules generated during cellular metabolism. Physiological levels of ROS participate in cellular signaling and the maintenance of homeostasis, whereas excessive ROS production or insufficient clearance can disrupt redox balance and aggravate cellular injury and inflammatory responses [59,60]. Antioxidant enzymes, including SOD and CAT play essential roles in limiting intracellular ROS accumulation by catalyzing the removal of superoxide anions and hydrogen peroxide. In the present study, treatment with the SMS ethanolic extract alone did not significantly alter intracellular ROS levels in RAW 264.7 macrophages, indicating that no evident pro-oxidant effect was observed within the tested concentration range. LPS stimulation significantly increased intracellular ROS levels, indicating an enhanced oxidative stress response in macrophages. Pretreatment with the SMS ethanolic extract markedly reduced LPS-induced ROS accumulation, restoring ROS levels to values close to those of the control group (Figure 11). Together with the partial recovery of SOD and CAT mRNA expression and the reductions in NO, TNF-α, IL-1β and IL-6, these findings indicate that SMS treatment was accompanied by concurrent attenuation of oxidative stress and inflammatory responses. Therefore, alleviation of LPS-induced ROS accumulation and redox imbalance may represent an important component of the inhibitory effect of SMS on inflammatory activation in RAW 264.7 macrophages.

#### 3.4.5. Effect of the Ethanolic Extract of SMS on the Protein Expression of Oxidative Stress Markers in LPS-Induced RAW 264.7 Macrophages

**Figure 12:**
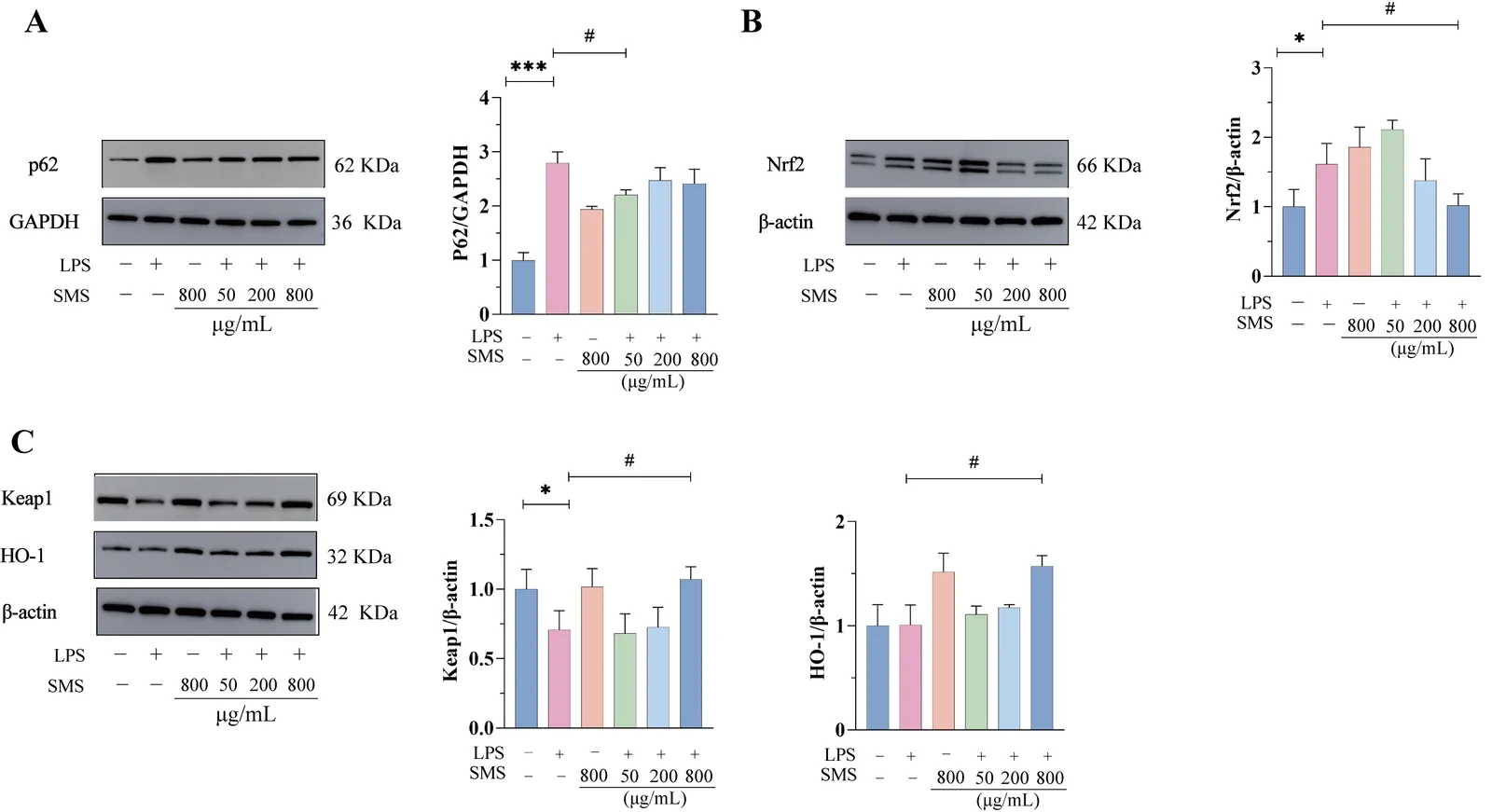
Effect of the ethanolic extract of SMS on the expression of p62, Keap1, Nrf2 and HO-1 proteins in LPS-induced RAW 264.7 macrophages (*n* = 3). (**A**) p62 protein expression and quantitative analysis; (**B**) Nrf2 protein expression and quantitative analysis; (**C**) Keap1 and HO-1 protein expression and quantitative analysis. −, without corresponding intervention/stimulation; +, with corresponding intervention/stimulation. All data are expressed as mean ± SD. * *p* < 0.05 and *** *p* < 0.001 indicate significant differences compared with the LPS-untreated control group; ^#^ *p* < 0.05 indicates significant differences compared with the LPS-induced group.

The p62/Keap1/Nrf2 regulatory system is an important mechanism through which cells respond to oxidative stress. As a key transcription factor, Nrf2 regulates the expression of numerous antioxidant and cytoprotective genes and contributes to the maintenance of cellular redox homeostasis and the regulation of inflammatory responses [61,62]. Under basal conditions, Keap1 promotes the ubiquitination and degradation of Nrf2. Upon oxidative or electrophilic stress, Keap1-mediated restriction of Nrf2 is weakened, allowing Nrf2 to become stabilized and translocate into the nucleus, where it regulates the transcription of antioxidant response element-related genes. p62 functions as both a selective autophagy receptor and a scaffold protein and can influence Nrf2 stability through its interaction with Keap1. Phosphorylation of p62 may further enhance its affinity for Keap1 and thereby participate in the regulation of cellular stress responses [63,64,65]. In the present study, LPS stimulation significantly increased p62 and total Nrf2 protein levels in RAW 264.7 macrophages while decreasing Keap1 protein expression, whereas HO-1 protein expression remained unchanged (Figure 12). These findings indicate that LPS markedly altered the expression profiles of p62-, Keap1- and Nrf2-related proteins. The LPS-induced accumulation of total Nrf2 may reflect a stress-associated response of macrophages to oxidative and inflammatory stimulation. However, the absence of a corresponding increase in HO-1 suggests that total Nrf2 abundance was not linearly associated with downstream protein expression. Following pretreatment with the SMS ethanolic extract for 6 h and subsequent LPS stimulation, 800 μg/mL SMS further increased p62 protein levels while decreasing total Nrf2 and increasing Keap1 expression. Previous mechanistic studies have demonstrated that p62 can competitively bind Keap1 and thereby reduce Keap1 mediated Nrf2 degradation. However, this process depends not only on total p62 abundance but also on the phosphorylation status of the Keap1 interacting region of p62. Site-specific phosphorylation can markedly enhance the interaction between p62 and Keap1, thereby promoting Nrf2 stabilization and the expression of downstream cytoprotective genes. Therefore, the concurrent increases in p62 and Keap1 and the decrease in total Nrf2 observed in the present study do not conform to a simple linear model in which p62 accumulation directly leads to Nrf2 activation. This pattern indicates that total p62 abundance alone may not adequately reflect the functional interaction between p62 and Keap1. p62 is also an important selective autophagy receptor that recognizes ubiquitinated proteins and participates in the autophagic clearance of protein aggregates. Its phosphorylation status can further influence the recognition and degradation of ubiquitinated substrates [66]. Accordingly, the further increase in p62 following SMS treatment may reflect alterations in the balance among p62 synthesis, autophagic degradation and the processing of stress-associated substrates. Meanwhile, the increase in Keap1 and decrease in total Nrf2 were directionally consistent with Keap1-mediated regulation of Nrf2 stability. Together with the reduction in intracellular ROS, these changes may reflect a decrease in LPS-induced oxidative stress burden and a corresponding attenuation of stress-associated Nrf2 accumulation. HO-1 exhibited a distinct response pattern. LPS stimulation alone did not significantly alter HO-1 protein expression, whereas combined treatment with 800 μg/mL SMS and LPS significantly increased HO-1 levels. Although HO-1 is an important cytoprotective protein regulated by Nrf2, its expression is not determined solely by total Nrf2 abundance. Mechanistic studies have identified BACH1 as an important physiological transcriptional repressor of the HO-1 gene. Heme and related cellular stress signals can reduce BACH1 occupancy at HO-1 regulatory regions, thereby permitting HO-1 transcription [67]. Therefore, the increase in HO-1 despite reduced total Nrf2 following SMS treatment may be associated with changes in Nrf2 subcellular localization or transcriptional activity, relief of BACH1-mediated transcriptional repression, or other stress-responsive regulatory mechanisms. Nrf2 has also been shown to restrict the transcription of pro-inflammatory genes, including IL-6 and IL-1β, in LPS-stimulated macrophages through mechanisms that are not entirely dependent on ROS removal or classical antioxidant response elements [68]. These findings further indicate that total Nrf2 abundance, nuclear transcriptional activity, HO-1 expression and inflammatory cytokine production are not necessarily linearly associated. Collectively, 800 μg/mL SMS-induced differential changes in p62, Keap1, total Nrf2 and HO-1, indicating a multilayered effect on oxidative stress-related proteins. Together with the reductions in intracellular ROS, restoration of SOD and CAT mRNA expression, and decreases in NO, TNF-α, IL-1β and IL-6, these findings suggest that SMS may contribute to the regulation of inflammatory responses in RAW 264.7 macrophages by alleviating the LPS-induced oxidative stress burden and altering the expression of proteins associated with cellular stress, proteostasis, and antioxidant defense.

## 4. Conclusions

Pretreatment with the SMS ethanolic extract attenuated LPS-induced inflammatory and oxidative stress responses in RAW 264.7 macrophages. These effects may be associated with reduced inflammatory mediator production, alleviation of cellular oxidative stress, and altered expression of p62, Keap1, total Nrf2 and HO-1 proteins. These findings provide an in vitro basis for further evaluation of the anti-inflammatory and antioxidant effects of SMS. However, because the present study examined a crude ethanolic extract, the principal bioactive constituents responsible for these effects remain incompletely characterized. Further studies are required to isolate, purify and identify the major active compounds, determine their relative contributions and potential interactions, and verify the underlying mechanisms and in vivo anti-inflammatory and antioxidant effects using targeted mechanistic interventions and appropriate animal models.

## Figures and Tables

**Table 1:** Sequences of primers used for polymerase chain reaction analysis.

Gene	Sequence of Primer (5′-3′)	Product Size	Gene Accession Number	Gene ID
IL-6	F: TGATGGATGCTACAAACTGGA	197 bp	NM_001314054.1	16193
	R: TGTGACTCCAGCTTATCTCTTGG			
IL-1β	F: TGCCACCTTTTGACAGTGATG	136 bp	NM_008361.4	16176
	R: ATGTGCTGCTGCGAGATTTG			
TNF-α	F: ACTGAACTTCGGGGTGATCG	107 bp	NM_001278601.1	21926
	R: CCACTTGGTGGTTTGTGAGTG			
IL-10	F: GCATGGCCCAGAAATCAAGG	162 bp	NM_010548.2	16153
	R: ACACCTTGGTCTGAGCTTATTA			
COX-2	F: TGGGGGAAGAAATGTGCCAA	161 bp	NM_011198.5	19225
	R: CAGCCATTTCCTTCTCTCCTGT			
iNOS	F: CAACAGGGAGAAAGCCAAAA	140 bp	NM_001313921.1	18126
	R: CAGGGATCTGAACATTCTGTG			
CAT	F: GCTCCGCAATCCTACACCAT	96 bp	NM_009804.2	12359
	R: GACATCAGGTCTCTGCGAGG			
SOD	F: GGGAAGCATGGCGATGAAAG	93 bp	NM_011434.2	20655
	R: GGTTCACCGCTTGCCTTCTG			
Nrf2	F: ACCTCTGCTGCAAGTAGCCT	118 bp	NM_001399226.1	18024
	R: TGGGCAACCATCACTCTGCT			
Keap1	F: GCCCCGGGACTCTTATTGTG	101 bp	NM_001110305.1	50868
	R: TTAGGGGCCCCGCCAT			
HO-1	F: GCTAGCCTGGTGCAAGATACT	60 bp	NM_010442.2	15368
	R: GCTGATCTGGGGTTTCCCTC			
GAPDH	F: GCCTCCTCCAATTCAACCCT	142 bp	NM_001289726.2	14433
	R: CCCAATACGGCCAAATCCGT			

Note: F, forward primer (5′-3′); R, reverse primer (5′-3′).

## Data Availability

The original contributions presented in this study are included in the article. Further inquiries may be directed to the corresponding authors.

## Abbreviations

ABTS	2,2-azino-bis-(3-ethyl-benzthia-6-ulfonic acid)
CCK-8	Cell counting kit-8
COX-2	Cyclooxygenase-2
DPPH	2,2-diphenyl-1-picrylhydrazyl
FBS	Fetal bovine serum
FRAP	Ferric-reducing antioxidant power
GAPDH	Glyceraldehyde-3-phosphate dehydrogenase
GSH	Glutathione
HO-1	Heme oxygenase-1
HPLC	High-performance liquid chromatography
IC_50_	Half-maximal inhibitory concentration
IL-1β	Iinterleukin-1beta
IL-6	Interleukin-6
IL-10	Interleukin-10
iNOS	Inducible nitric oxide synthase
Keap1	Kelch-like ech-associated protein 1
LDH	Lactate dehydrogenase
LPS	Lipopolysaccharide
MDA	Malondialdehyde
NO	Nitric oxide
Nrf2	Nuclear factor erythroid 2-related factor 2
P62	Sequestosome 1
qRT-PCR	Quantitative reverse transcription polymerase chain reaction
ROS	Reactive oxygen species
SD	Standard deviation
SM	*S* *ophora moorcroftiana*
SMS	*Sophora moorcroftiana* seeds
SOD	Superoxide dismutase
TNF-α	Tumor necrosis factor-alpha
TPTZ	2,4,6-tripyridyl-s-triazine
VC	Ascorbic acid
